# Production and characterization of feldspathic-muscovite glass composite for dental prosthesis

**DOI:** 10.1590/1807-3107bor-2025.vol39.066

**Published:** 2025-08-04

**Authors:** Fernanda Paes de Figueiredo Costa PIETOSO, Shirleny Fontes SANTOS, Lucas Hian da SILVA, Paulo Francisco CESAR

**Affiliations:** (a) Universidade Federal Fluminense – UFF, School of Dentistry, Niterói, RJ, Brazil.; (b) Fundação Centro Universitário da Zona Oeste – UEZO, Department of Materials Engineering, São Paulo, SP, Brazil.; (c) Faculdade Israelita de Ciências da Saúde Albert Einstein – FICSAE, School of Dentistry, São Paulo, SP, Brazil.; (d) Universidade de São Paulo – USP, School of Dentistry, Department of Biomaterials and Oral Biology, São Paulo, SP, Brazil.

**Keywords:** Ceramics, Dental Porcelain, Glass, Dental Prosthesis

## Abstract

The principal objective of this studywas to produce and characterize a machinable glass ceramic containing muscovite-mica as the main crystalline phase to be used as a dental restorative material. The secondary objective was to evaluate the use of muscovite-mica to improve machinability and generate a toughening mechanism in the experimental glass ceramic. After fine milling of a feldspathic glass frit was milled and then mixed with muscovite-mica, die-pressed, and sintered under vacuum at 850 to 1,150 °C. The resulting sintered composite was characterized by X-ray diffraction, scanning electron microscopy, and had its fracture toughness evaluated by micro-indentation. The results were as follows: (a) improved thermal stability of muscovite-mica crystals in the composite; (b) improved leucite crystallization in the feldspathic glass matrix by increasing sintering temperature in the studied range ; (c) the composites consisting of glass + 10% muscovite and glass + 20% muscovite sintered at 1,050°C presented fracture toughness values of 9.0 ± 1.2 and 8.4 ± 0.6 MPa.m^1/2^, respectively, which are higher than those found in the literature for glass ceramics. Feldspar frit blocks with addition of muscovite-mica (up to 20%) can be densified at temperatures between 1,050 and 1,150°C. This material was stable at a temperature substantially higher than the stability limit of pure muscovite and also showed indentation fracture toughness values greater than those reported in the literature for other glass ceramics.

## Introduction

Ceramic restorations are aesthetically pleasing compared to other currently available restorative materials . These restorations are produced by processing techniques so that they can mimic the natural tooth in terms of color, surface texture, and translucency. With the increasing demand for aesthetics among patients, these restorations have become an important part of contemporary dental practice.^1^

Dental ceramics have excellent properties to be used as dental restorative materials, such as high mechanical strength, high chemical stability, high biocompatibility, and high wear resistance. These properties make ceramics one of the most popular group of materials among clinicians and patients.^2,3^There are several types of processing methods to produce all-ceramic dental restorations, such as hot-pressing, glass infiltration in partially sintered ceramics, machining of ceramic blocks, and the stratification technique.^4^ Machinable ceramics can be processed in CAD-CAM systems to produce inlays, onlays, crowns, fixed partial dentures, and veneers.

One of the first machinable glass-ceramic blocks used to produce dental restorations contained tetrasilicic fluormica- glass as the crystalline phase (Dicor MGC, Corning Inc., Corning N.Y). However, this material was discontinued by the manufacturer because of low tensile strength, the competition with ceramics that have excellent mechanical and optical properties (e.g., VITA ceramics), and the need to color the restoration in the external region rather than in the core region. The good machinability of Dicor MGC was associated with the fact that tetrasilicic fluormica- crystals were easily worn down by diamond burs. Mica is a generic term used to describe the group of complex aluminosilicate minerals that have a lamellar structure. This structure enhances the mechanical strength by impeding the propagation of cracks within their unique structure where its atoms are arranged in thin layers that can interrupt the path of cracks.^5,6^

The mica group comprises several known minerals such as biotite, phlogopite, muscovite, among others.^7^

The present study proposed the use of muscovite-mica to improve the machinability and generate toughening mechanisms in experimental glass ceramics. This type of mica is found in nature and is present on a large scale in the Brazilian territory, making it a low-cost and easily obtainable material . Also, the environmental impact caused by its disposal in nature is low. Thus, the objective of this study was to produce and characterize a machinable glass ceramic containing muscovite-mica as the main crystalline phase to be used as a dental restorative material.

## Methods

### Material

The basic experimental glass-ceramic (control) composition used in this study is shown in Table 1. This composition was chosen because it can be melted at 1,200°C and produce a high concentration of leucite crystals, which provide excellent mechanical resistance to the material. The main raw material used for the production of the frits of this glass ceramic was the Mineiração Armil feldspar, from the Borborema-Seridó (region between the states of Paraíba and Rio Grande do Norte, Brazil), to which appropriate amounts of alumina (0.53 g), sodium (1.56 g), and potassium carbonates (1.74 g), borax (0.5 g), and cerium oxide (0.13 g) were added. The chemical composition of Armil feldspar is described in Table 2. This material was dry-milled in a blender, predominantly in a particle size range between 12 and 50 μm, considered suitable for the intended use.

**Table 1:** Composition of the glass ceramic used in the present study.

Components	% by weight
SiO_2_	56.1
Al_2_O_3_	19.9
CeO_2_	0.7
K_2_O	11.2
Na2O	10.5

**Table 2:** Chemical composition of Armil feldspar.

Components	% by weight
SiO_2_	66
Al_2_O_3_	20
Fe_2_O_3_	0.07
K_2_O	5.5
Na2O	5.9

### Specimen manufacture

The raw materials for frit production were weighed to 20 g on a batch precision scale. After weighing the raw materials, the powders were homogenized in a ball mill for 24 h. Once homogenized, the powder was poured into a refractory crucible and muffled at 1,200°C for three hours for melting to occur, then abruptly cooled to vitrify the material.

The ground chips were mixed in the following proportions of muscovite-mica: 0% (pure frit), 10% mica, 20% mica, and 100% (pure mica). For the production of the blocks, two matrix sizes were used: a stainless steel cylindrical matrix with 16 mm of internal diameter (for X-ray diffraction analysis) and a smaller cylindrical matrix with 6 mm of internal diameter (intended for SEM analysis). For cold pressing, the largest matrix was used with a hydraulic pressure of 5,600 pounds, and for the smallest matrix, a pressure of 500 pounds was used. For each large block, 4 g of the mixture of each material was used, and for the small block, approximately 0.3 grams was used.

Sintering was performed at seven different temperatures: 850°C, 900°C, 950°C, 1,000°C, 1,050°C, 1,100°C, and 1,150°C. For each sintering temperature, two blocks of each material mixture were produced, a smaller one for scanning electron microscopy (PEMM/COPPE/UFRJ and CETEM/MCT), and a larger block for X-ray diffractometry (CETEM/MCT). Table 3 shows the temperatures at which each material mixture was sintered. The automatically programmed furnace heating rates are also shown in the table. All sintering cycles were carried out under vacuum using a VACUMAT 40 (Vita) furnace.

**Table 3:** Sintering conditions for each different material mixture.

Variable	850°C	900°C	950°C	1.000°C	1.050°C	1.100°C
	20°C/min	22°/min	23°C/min	25°C/min	26°C/min	28°C/min
Frit	X	X				
Frit + 10% mica	X	X	X	X	X	X
Frit + 20% mica	X	X	X	X	X	X
Mica	X	X	X	X	X	X

### Scanning electron microscopy (SEM) and X-ray diffraction (XRD) analysis

The microstructure of the silver-coated sintered blocks was analyzed under a scanning electron microscope (Leica model F440) in the high vacuum mode.

The crystallographic structures of the pure frit, with 10% muscovite-mica, 20% muscovite-mica, and pure muscovite-mica powders subjected to different sintering temperatures were analyzed. Pure frit specimens were also analyzed before sintering to verify whether the specimen was amorphous. These analyses were determined using a BRUKER-AXS D5005 diffractometer, CoKα radiation (35 kV / 40 mA); 2θ per step, with a time count of 1 s per step, and collected at 5 to 80º 2θ. The qualitative interpretation of the spectrum was made by comparison with standards from the PDF02 database (ICDD, 2006) in Bruker DiffracPlus software.

### Hardness and elastic modulus measurements

Vickers hardness and elastic modulus values for the 10% sintered mica frit at 1,050°C and the 20% sintered mica frit at 1,150 °C were determined by the indentation method. The tests were performed with the help of an indenter (CSM Instruments S.A. version R0.0.5, 2006), and the Oliver & Pharr Method was used. A sample of each material was prepared. Both were rectified, polished, and gold-sputtered to facilitate microscope visualization. Three indentations were made at different locations on each specimen, using a load of 0.5 kg. Vickers microhardness and modulus of elasticity values were calculated by the software. The elastic modulus is given by the following equations (1):


$$E_{IT}=\frac{1-V_{S}^{2}}{\frac{1}{E_{r}}-\frac{1-V_{i}^{2}}{E_{i}}}\text{ with }E_{r}=\frac{\sqrt{\pi \cdot S}}{2\cdot \beta \cdot \sqrt{A_{p}\left(h_{c}\right)}}$$ (1)

Equations were applied using the software to calculate the values of the modulus of elasticity, EIT and Er (Indentation software User’s Guide 2006, CSM Instruments S.A.). Where: Ei = Indenter modulus of elasticity (1,141 GPa), νi = Indenter’s Poisson ratio (0.07), Er = Reduced indentation contact modulus and νs = Poisson ratio of the sample (0.21).

To calculate Vickers hardness, the software (Indentation software User’s Guide 2006) used the following equation (2):


$$A_{p}=f(h)$$ (2)

A_*p*_: Projected contact area (theoretical or calibrated)


$$H_{IT}:H_{IT}=\frac{F_{\max}}{A_{p}\left(h_{c}\right)}\text{ in Pascal }$$

$$H_{V}:H_{V}=\frac{F_{\max}}{9.81\cdot A_{c}\left(h_{c}\right)}$$

### Fracture toughness measurements

The indentation method was used to calculate fracture toughness (K_Ic_). Eight specimens of the muscovite-mica glass ceramic were prepared: four specimens with 10% sintered mica at 1,050°C and four specimens with 20% sintered mica at 1,150 °C. Each specimen was prepared so that the indentation surface was parallel to its base. After that, the surface was polished and gold-sputtered to allow for visualization under the optical microscope. Ten indentations were performed in different regions of the specimens with the aid of a Leitz Durimet 2 microdurometer with a load of 2 kgf for 30 s. A digital camera (NIKON, Coolpix-950) was attached to the microhardness eyepiece, thus obtaining the photomicrographic record of the indentations immediately after the removal of the indenter. Through these records, it was possible to evaluate the correct indentations for fracture toughness measurements, that is, four radial cracks from the center of the indentations, free of branches or chips.

Fracture mechanics analysis indicates that the length of the radial crack, c, produced by a Vickers indentation, is inversely related to K_Ic_ by the following correlation: K_Ic_ ∝ c – 3/2.^8^

Anstis et al.^9^ studied the application of Vickers indentation techniques to evaluate the fracture toughness of ceramic materials. To calculate the K_Ic_ value, the authors developed the following equation (3):


$$K_{\mathrm{Ic}}=0.016(E/H{)}^{1/2}(P/c3/2)$$ (3)

where: P = indentation load (N); E = Young’s modulus (modulus of elasticity) (GPa) H = Vickers Hardness = Mean radial crack measurements (m). Equation 3 was used to determine the fracture toughness of the two materials chosen: frit with 10% muscovite-mica sintered at 1,050°C and frit with 20% muscovite-mica sintered at 1,150°C.

## Results

**Figure 1:**
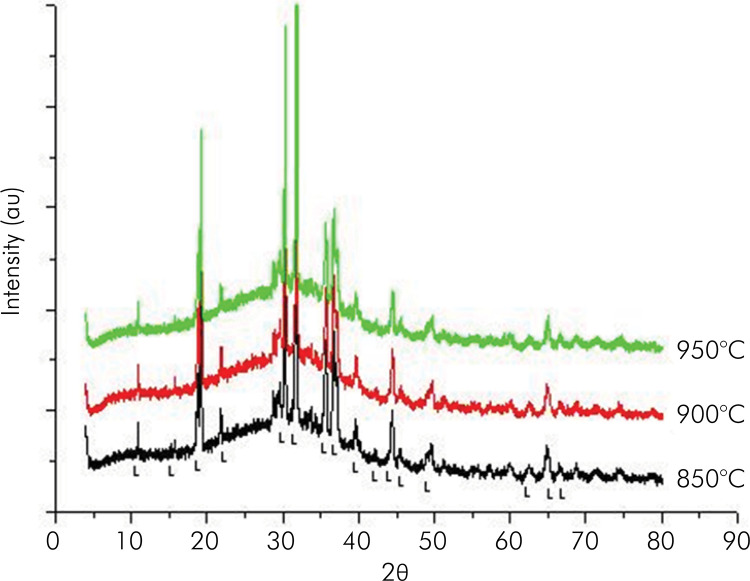
Diffractograms of the sintered pure frit block at temperatures of 850, 900 and 950°C. (Peaks identified with L = Leucite).

**Figure 2:**
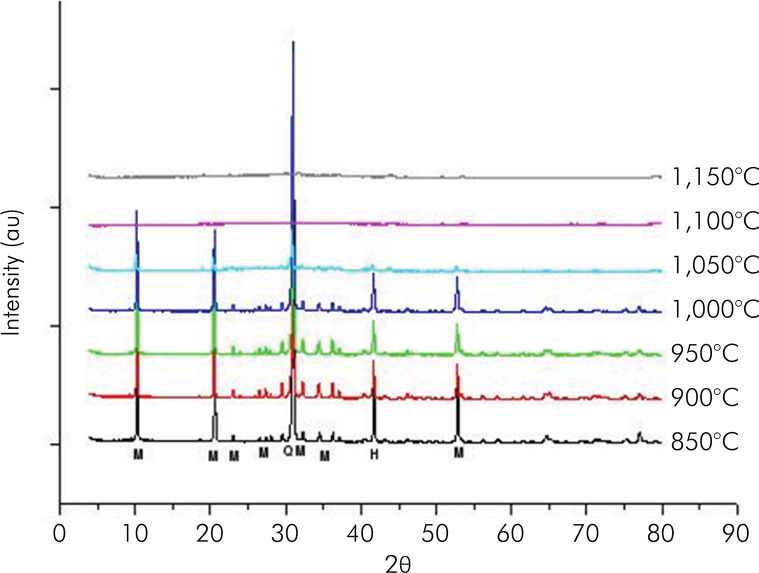
Diffractograms of sintered pure muscovite mica blocks at temperatures in the range of 850 to 1,050°C (Peaks identified with M = Muscovite mica; QM = quartz; H = Hematite).

Figure 1 shows the diffractograms of the sintered pure frit block at temperatures of 850, 900, and 950°C. Note that the sizes of the most intense leucite peaks increased as the sintering temperature was raised (range of 850 to 950°C). Figure 2 shows the diffractograms of sintered pure muscovite-mica blocks at temperatures in the range of 850 to 1050 °C. These results indicate that pure muscovite-mica is not stable at temperatures exceeding 1,000°C, as evidenced by the graph at the temperature of 1,050°C, which showed a decrease in muscovite peaks, due to the presence of amorphous material. Possibly, pure muscovite-mica gradually decomposed into feldspar glass.

**Figure 3:**
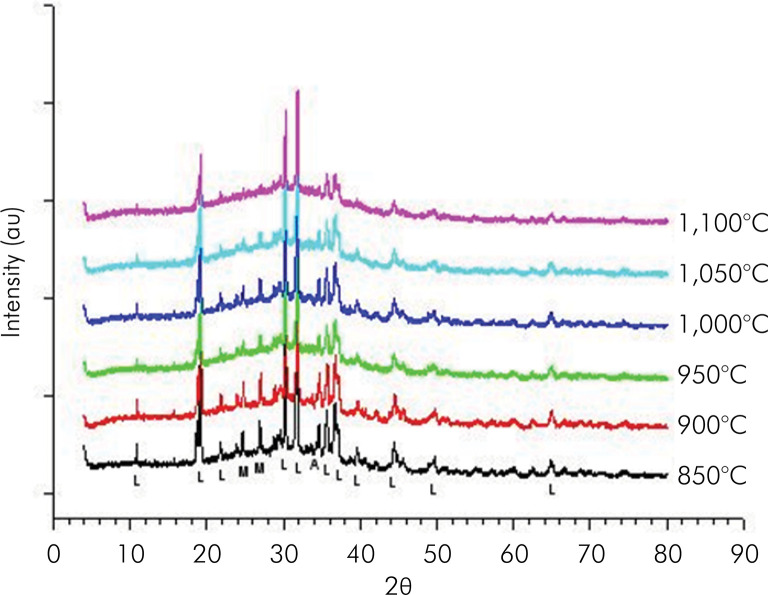
Diffractograms of glass frit blocks with 10% addition of Muscovite mica and sintered at temperatures from 850 to 1100 °C (Peaks identified with M = Muscovite mica; L = Leucite; A = Alumina).

Figure 3 shows the X-ray diffractograms of glass frit blocks with 10% addition of muscovite-mica and sintered at temperatures from 850 to 1,100°C. The sizes of the most intense peaks of leucite gradually increased as the sintering temperature was raised. In contrast, the most intense peaks of muscovite-mica decreased in the same temperature range. At 1,100°C, only remnants of the muscovite-mica phase remained.

**Figure 4:**
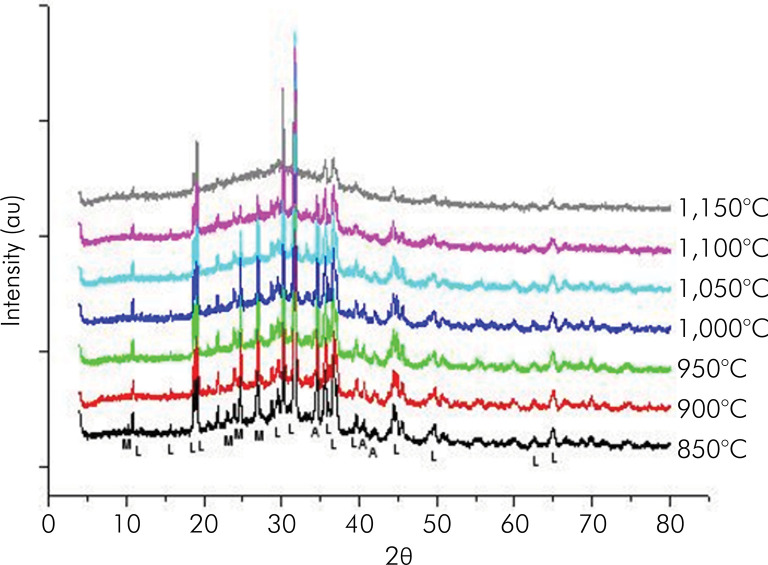
Diffractograms of glass frit blocks with the addition of 20% sintered muscovite mica at temperatures ranging from 850 to 1150 °C. (Peaks identified with M = Muscovite mica; L = Leucite; A = Alumina).

Figure 4 shows the X-ray diffractograms of glass frit blocks with the addition of 20% sintered muscovite-mica at temperatures ranging from 850 to 1,150°C. The most intense peaks of leucite gradually increased as the sintering temperatures were raised. In contrast, muscovite-mica peaks were restricted to only remnants of the sintered product at 1,150°C.

**Figure 5:**
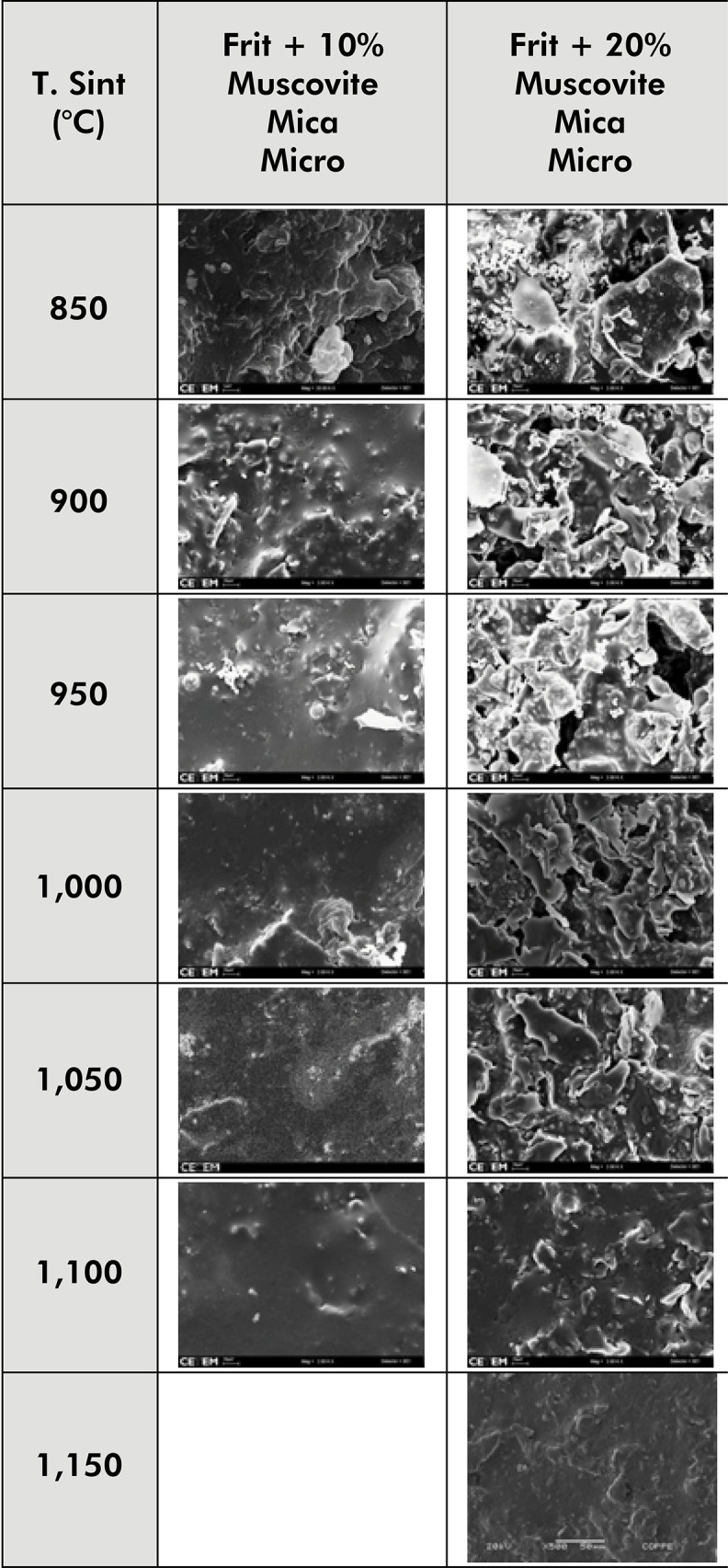
Photomicrographs of frit blocks with 10% and 20% Muscovite mica at different sintering temperatures. The images show the densification of the material as sintering temperatures increase, with the glass matrix becoming more continuous and allowing the Muscovite grains to integrate with virtually no porosity.

Figure 5 shows the microstructural characteristics of frit blocks with 10% and 20% muscovite-mica. This figure shows that at a sintering temperature of 1,050°C or higher, the block containing 10% muscovite-mica has a dense microstructure because of the continuity of the glass matrix where the muscovite grains integrate virtually without porosity. Blocks containing 20% muscovite-mica only acquire such a dense microstructure at a sintering temperature of 1,150°C.

Table 4 shows the elastic modulus (E), hardness (H), and apparent fracture toughness (K_Ic_) of glass frit blocks + 10% sintered muscovite-mica at 1,050°C and glass frit + 20% sintered muscovite-mica at 1,150°C. There was no significant difference between these two experimental groups regarding any of the measured properties.

**Table 4:** Mean of elastic modulus (E), hardness (H), and apparent fracture toughness (KIc) of glass frit blocks + 10% sintered muscovite-mica at 1050 °C and glass frit + 20% sintered muscovite mica at 1,150°C (standard deviations in brackets).

Mica concentration (%)	E (GPa)	H(GPa)	K_Ic_ (MPa.m^1/2^)
10%	66.2 (0.9)^a^	8.6 (0.1)^a^	9.0 (1.2)^a^
20%	65.0 (0.4)^a^	8.5 (0.1)^a^	8.4 (0.6)^a^

Different letters represent statistical difference at a 5% level by the Student’s *t*-test.

## Discussion

Mica-based glass ceramics have a microstructure with a percentage of 50% to 70% by volume of mica dispersed in a glass matrix.^10^ The mica crystals are elongated and randomly oriented, and because of their arrangement in the matrix, they serve as crack deflectors, increasing the toughness of the material.^5,6^ This information indicates that it would be desirable to increase the muscovite-mica content of the mixture (feldspar glass frit + muscovite-mica) beyond the 20% limit considered in the present study.

By analyzing the macroscopic images of the feldspar glass plus mica-muscovite frit blocks, it was found that they could only maintain external macroscopic integrity at sintering temperatures of 1,100°C and 1,150°C. The muscovite-mica content was higher than 10%, and the internal densification was best for 20% mica sintered at 1,100°C.

The dispersion of muscovite-mica crystals in the leucite-reinforced feldspar glass matrix allowed for the stabilization of muscovite-mica at high temperatures because the occlusion provided by feldspar glass kinetically hindered the release of mica decomposition in water. This fact suggests that it is possible to produce leucite-reinforced feldspar glass composites associated with muscovite-mica in contents higher than the 20% used in the present study, and this composition may achieve better results in terms of mechanical properties.

The present study showed that the decomposition of mica inevitably occurs around 1,100°C to 1,150°C, as can be observed in the sintered block diffractograms of materials containing 10% to 20% muscovite-mica.

Pure muscovite mica is not stable at temperatures above 1,050°C. The only peak present in sintered specimens at 1,100°C and 1,150°C is that of quartz, which already appears in the sintered specimen at 850°C. This means that pure muscovite-mica gradually decomposes into quartz and feldspar glass, as shown in previous studies. Zhang et al.^11^ mixed fluormica crystals (35%) with powdered glass (65%) and evaluated their properties. X-ray diffraction analyses showed that the fluormica crystals were unstable at temperatures above 900 °C, and were transformed into other phases, showing that other types of mica also degrade at high temperatures.

The X-ray diffractograms of sintered glass frit blocks containing 10% muscovite-mica sintered at temperatures from 850°C to 1,100°C revealed in that the sizes of the most intense leucite peaks gradually increased as the sintering temperature was raised. In contrast, the most intense peaks of muscovite-mica decreased in the same temperature range, with only remnants of this phase at 1,100°C. Therefore, the temperature of 1,050°C was the maximum sintering temperature for this material, at which a detectable amount of muscovite-mica was detectable in the final material.

Similarly, the X-ray diffractogram of blocks containing 20% of muscovite-mica sintered at temperatures ranging from 850°C to 1,150°C showed that the most intense leucite peaks gradually increased as the sintering temperature was raised. However, muscovite-mica peaks were restricted to only remnants in the sintered product at 1,150°C. This fact indicated that 1,150°C is the ideal sintering temperature so that the final glass ceramic still had a detectable amount of muscovite-mica phase.

The K_Ic_ results of blocks containing 10% or 20% of muscovite-mica and sintered at 1,050°C showed that these two products had equivalent resistance to crack propagation. Thus, the present study was able to obtain a relatively tough glass ceramic (feldspar glass frit + muscovite-mica), which can be sintered at modest temperatures varying from 1,050°C to 1,150°C.

Quin et al.^12^ concluded that the indentation technique is not reliable as a fracture toughness test for ceramics because these measurements in terms of fracture resistance can not be readily defined. However, despite the disadvantages of this method, Cesar et al.^13^ also highlighted that the indentation technique is considered simple, fast, and requires very little material for testing. Due to the non-destructive nature of the test and the minimal need for experimental materials, it was possible to perform it in this study.

Yoshimura et al.^14^ evaluated fracture toughness of six dental porcelains with leucite content by indentation fracture (IF), surface crack in flexure (SCF), and single edge pre-cracked beam (SEPB) methods. The K_Ic_ value obtained by the indentation method for porcelain with 22% leucite was 0.9 MPa.m^1^, which is lower than the experimental ceramics in this study.

New studies need to be developed with these experimental materials to compare them with other commercial materials. For instance, using another method for testing fracture toughness that provides more reliable results according to the literature.

## Conclusion

Feldspar frit blocks with addition of muscovite-mica (up to 20%) can be densified at temperatures between 1,050°C and 1,150°C. This material was stable at a temperature substantially higher than the stability limit of pure muscovite and also showed indentation fracture toughness values above those reported in the literature for other glass ceramics.

## Data Availability

The datasets generated during and/or analyzed during the current study are available from the corresponding author on reasonable request."

## References

[1] Rosenstiel J, Land SF, Fujimoto MF (2002). Prótese fixa contemporânea.

[2] Sun Z, Zhou Y, Wang J, Li M (2007). ?-Y2Si2O7, a machinable silicate ceramic: mechanical properties and machinability. J Am Ceram Soc.

[3] Thompson JY, Bayne SC, Heymann HO (1996). Mechanical properties ceramic for CAD / CAM of a new mica-based restorations. J Prosthet Dent.

[4] Al-Shammery CC, Wood HA, Bubb DJ, Youngson NL (2004). Novel machinable mica based glass ceramics for dental applications. Glass Technol.

[5] Gali S (2019). Mica glass ceramics for dental restorations. Materials Technology.

[6] Gali S, Chiru SR (2021). Machinability of zirconia toughened mica glass ceramics for dental restorations. Braz Dent Sci.

[7] Baltar PMT, Sampaio CAM, Cavalcante JA, Baltar PMT, Sampaio CAM, Cavalcante JA (2005). Mica.

[8] Lawn B, Wilshaw R (1975). Indentation fracture: principles and applications. J Mater Sci.

[9] Anstis GR, Chantikul P, Lawn BR, Marshall DB (1981). A critical evaluation of indentation techniques for measuring fracture toughness: I, Direct Crack Measurements. J Am Ceram Soc.

[10] Craig J, Powers RG (2004). Materiais dentários restauradores.

[11] Zhang WY, Gao H, Li BY, Jiao Q. Q., Jiao QB. (2006). A novel route for fabrication of machinable fluoramphibole glass-ceramics. Scr Mater.

[12] Quinn GD, Bradt RC (2007). On the Vickers indentation fracture toughness test. J Am Ceram Soc.

[13] Cesar PF, Della Bona A, Scherrer SS, Tholey M, Noort R, Vichi A (2017). ADM guidance-Ceramics: fracture toughness testing and method selection. Dent Mater.

[14] Yoshimura HN, Cesar PF, Miranda WG, Gonzaga CC, Okada CY, Goldenstein H (2005). Fracture Toughness of Dental Porcelains Evaluated by IF, SCF, and SEPB Methods. J Am Ceram Soc.

